# Proteome Profiling of Cerebral Vessels in Rhesus Macaques: Dysregulation of Antioxidant Activity and Extracellular Matrix Proteins Contributes to Cerebrovascular Aging in Rhesus Macaques

**DOI:** 10.3389/fnagi.2019.00293

**Published:** 2019-10-23

**Authors:** Xia Wang, Yifan Liu, Yangjie Jia, Haotian Liu, Xinjie Bao, Zhanlong He, Wei Ge

**Affiliations:** ^1^State Key Laboratory of Medical Molecular Biology, Department of Immunology, Institute of Basic Medical Sciences, Chinese Academy of Medical Sciences, School of Basic Medicine, Peking Union Medical College, Beijing, China; ^2^Department of Neurosurgery, Peking Union Medical College Hospital, Chinese Academy of Medical Sciences, Peking Union Medical College, Beijing, China; ^3^Yunnan Key Laboratory of Vaccine Research and Development on Severe Infectious Disease, Institute of Medical Biology, Chinese Academy of Medical Sciences, Peking Union Medical College, Kunming, China; ^4^Department of Neurosurgery, Affiliated Hospital of Hebei University, Baoding, China

**Keywords:** aging, rhesus macaque, cerebral vessels, proteomics, reactive oxygen species, extracellular matrix

## Abstract

Aging is a major risk factor for cerebrovascular disease; however, the molecular mechanisms of cerebrovascular aging remain to be clarified. The aim of this study was to reveal the molecular signaling pathways involved in cerebrovascular aging. This study used high-resolution liquid chromatography coupled to tandem mass spectrometry (LC-MS/MS), in combination with quantitative 6-plex tandem mass tag labeling, to profile protein changes in brain vessels from three groups of healthy rhesus macaques (3-years, 6-years, and 20-years). Western blot analyses were used to validate the proteomic data. A total of 2,934 proteins were identified and analyzed. Twenty-two proteins were continuously downregulated with increasing age, while three proteins were continuously upregulated. When comparing Group C vs. Group B, 270 proteins were downregulated, while 73 proteins were upregulated. All these 368 significantly changed proteins were used for further analysis. Bioinformatic analysis showed that the changed proteins were involved in several signaling pathways during cerebrovascular aging. Proteins in the NRF2 pathway, such as Glutathione S-transferase Mu (GSTM), were consistently downregulated especially after 6-years old, whereas proteins related to miRNA targets in the extracellular matrix (ECM) and membrane receptors were upregulated. Protein-protein interaction networks demonstrated that disorders of energy pathways and serine/threonine kinases were critical during cerebrovascular aging. Data are available *via* ProteomeXchange under the identifier PXD012306. Our results indicated that during aging, the disorders of energy metabolism and dysfunction of antioxidant activity caused over-production of reactive oxygen species (ROS) may exacerbate cerebrovascular aging. In addition, accumulation of ECM proteins during aging might be closely associated with age-related arterial stiffening and decreased compliance.

## Introduction

Aging is an enormous challenge worldwide. Currently, approximately 177 million people in China are aged >65 years and it is estimated that more than 30% of the Chinese population, equating to 390 million people, will be aged >65 years by 2050. Age-related diseases are a serious problem in China (Wang et al., 2017). Aging is the major risk factor for a series of cardiovascular and cerebrovascular diseases, such as hypertension and stroke (Wang et al., 2017). Although many studies have focused on age-related diseases, few have investigated the molecular changes during cerebrovascular aging.

Vascular aging is the outcome of structural and functional changes in the vascular endothelium and the arterial wall caused by oxidative stress (Donato et al., 2007; Valko et al., 2007; Ungvari et al., 2010a; Izzo et al., 2018) as well as changes in the structure of extracellular matrix (ECM; Zieman et al., 2005; Izzo et al., 2018). Oxidative stress is a pathological condition which is the outcome of the disequilibrium between the production and elimination of reactive oxygen species (ROS). NRF2 pathway has antioxidative activity, and the suppression of the NRF2 pathway seems to be the critical process in ROS-mediated vascular dysfunction (Ungvari et al., 2008, 2011a,b). The increased rate of programmed cell death of vascular smooth muscle cells and the altered expression of collagen and elastin in the ECM also increases vascular stiffness during aging (Lacolley et al., 2017).

Proteomics is a global, unbiased and hypothesis-free approach to depicting the status and dynamic changes in specific tissue. Proteomics can be used to identify the key molecules and pathways in diverse physiological and pathological processes (Zhu and Snyder, 2002). The application of quantitative proteomics is becoming increasingly common in neurological studies of aging and quantitative protein profiling of the hippocampus (Xu et al., 2016a) and temporal lobe (Xu et al., 2016b) in the brain during human aging has been reported. However, proteomics analysis of the changes in protein expression in cerebral vessels during aging has not yet been conducted.

Demetrius reported that mice and humans differ significantly in terms of morphology, physiology and life history. Simple extrapolation from mouse models to human systems in studies of the aging process may be invalid (Demetrius, 2005). Shi et al also reported that due to the vast dissimilarities in brain size and structure between humans and rodents, the traditional mouse and rat models are less than ideal as models for human brain evolution research (Shi et al., 2019). Rhesus macaque monkeys (Macacamulatta) show high sequence similarity with human (>93% for protein-coding genes) and are regarded as a preferable animal model (Yan et al., 2011).

Thus, in this study, we conducted TMT-labeled quantitative proteomic analysis of cerebral vessels from rhesus macaques collected during aging. Bioinformatic analysis revealed the changes in protein expression and pathways related to cerebrovascular aging. Our results showed that in the aging process, disorders of energy metabolism and dysfunction of antioxidant activity cause over-production of ROS and may exacerbate cerebrovascular aging. Furthermore, accumulation of ECM proteins during aging might be closely associated with age-related arterial stiffening and decreased compliance.

## Materials and Methods

### Reagents

Urea was purchased from GE Healthcare (LC, UK). Proteinase inhibitor cocktail was purchased from Roche (BS, CH). TMT (Tandem Mass Tag^TM^) kits were purchased from Thermo Fisher Scientific (NJ, USA). Sequencing grade Trypsin/Lys-C was purchased from Promega (WI, USA). Antibodies: Anti-ACTB (GTX124213, GeneTex, CA, USA), anti-FN1 (ab32419, Abcam, Cambridge, UK), and anti-NOS3 (sc-376751, Santa Cruz, CA, USA). The other reagents were obtained from Sigma (MO, USA).

### Sample Collection and Preparation

In this study, the cerebral vein specimens of healthy rhesus macaques were obtained from the Institute of Medical Biology, Chinese Academy of Medical Sciences and Peking Union Medical College (Beijing, China). Accordingly, rhesus macaques were humanely euthanized by anesthesia with ketamine HCl (10 mg/kg) followed by sodium pentobarbital overdose. After deep anesthesia, the rhesus monkeys were killed, brain tissues were isolated and cerebral veins were dissected. The current study was approved by the Ethical Review Committee of the Chinese Academy of Medical Sciences.

In this study, cerebral veins were resected from brain cortex of nine healthy rhesus macaques. No senile plaques were detected in these rhesus brain tissues (Supplementary Figure S1). Vessel specimens were divided into three groups: Group A (age = 3 years, two males and one female), Group B (age = 6 years, one male and two females), and Group C (age = 20 years, two males and one female). All vessel specimens were rinsed thoroughly and cut into small pieces in ice-cold PBS. The treated specimens were then centrifuged (12,000 rpm, 3 min) to remove residual blood.

### Protein Extraction and TMT Labeling

Specimens were immediately homogenized in liquid nitrogen, incubated with lysis buffer (8 M urea in PBS (pH 8.0), 1× protease inhibitor cocktail, 1 mM phenylmethanesulfonyl fluoride) at 4°C for 40 min. Lysates were clarified by centrifugation (12,000 *g*, 15 min, 4°C). Protein concentration in the supernatants was measured spectrophotometrically (280 nm) using NanoDrop 2000. Equal amounts of protein (100 μg) from each group were pooled. Proteins were treated with 10 mM dithiothreitol for 0.5 h at 37°C, and then incubated with 25 mM iodoacetamide for 30 min in the dark at room temperature. The proteins were diluted with PBS (pH 8.0) to a final urea concentration of 1.0 M and digested with Trypsin/Lys-C (4 μg) overnight at 37°C. The digested peptides were desalted before the addition of 50 μl 200 mM triethylammonium bicarbonate buffer. The peptides were then labeled with different TMT labels: Group A labeled with TMT-127, Group B labeled with TMT-129, and Group C labeled with TMT-130. The labeled peptides from the three groups were then mixed for further MS analysis.

### HPLC Fractionation

HPLC analysis of the mixed peptides was performed according to a previously described method (van Ulsen et al., 2009). The peptides were fractionated by gradient elution (mobile phase A: H_2_O; mobile phase B: 98% acetonitrile, both adjusted to pH 10 with ammonium hydroxide) using an UltiMate 3000 UHPLC (Thermo Fisher Scientific, NJ, USA) fitted with an Xbridge BEH300 C18 column (4.6 mm × 250 mm, 5 μm, 300 Å, Waters). The following gradient was used: 5%–8% mobile phase B (0–5 min), 8%–18% mobile phase B (5–40 min), 18%–32% mobile phase B (40–62 min), 32%–95% mobile phase B (62–64 min), 95% mobile phase B (64–72 min). Starting at 2 min, fractions (*n* = 47) were collected at 1.5 min intervals and dried under vacuum before being combined into 12 fractions. Peptides were then dissolved in 20 μl 0.1% TFA for LC-MS/MS analysis.

### LC-MS/MS Analysis

Peptides were fractionated for LC-MS/MS analysis with the Ultimate U3000 system using a silica capillary column (75 μm ID, 150 mm length; Upchurch, Oak Harbor, WA, USA) packed with C-18 resin (300 Å, 2 μm; Varian, Lexington, MA, USA). The separation was achieved with a 135 min gradient elution of mobile phase A (0.1% formic acid) and mobile phase B (100% acetonitrile and 0.1% formic acid) set at a flow rate of 0.3 μl/min. Detected fractions were analyzed using Xcalibur 4.1 software. Data were acquired using the directly interfaced Orbitrap Fusion mass spectrometer (operated in the data-dependent acquisition mode) with a single full-scan mass spectrum (350–1,550 m/z, 120,000 resolution) followed by data-dependent MS/MS scans (3 s) in an Ion Routing Multipole at 35% normalized collision energy (HCD).

### Bioinformatics Analysis

**Figure 1:**
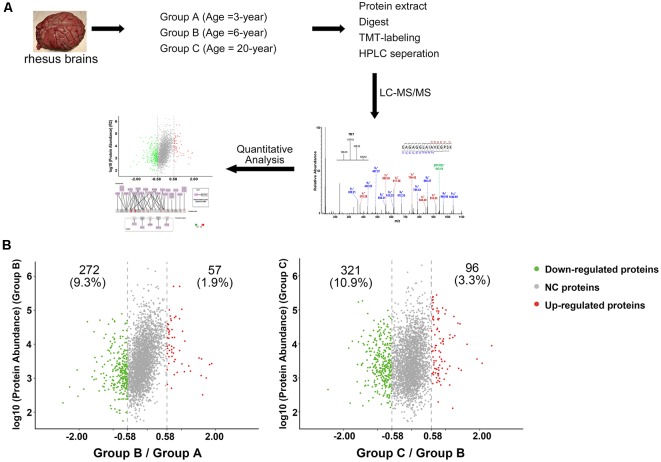
Characterization of protein profiles in cerebral vessels. **(A)** Experimental workflow for protein profiling of cerebral vessels. ** (B)** Abundance ratio of proteins are depicted when comparing Group B to Group A and Group C to Group B. Proteins with an abundance ratio <0.67 or >1.50 were defined as significantly changed proteins. Downregulated proteins are represented as green dots; upregulated proteins are represented as red dots; other proteins were defined as non-significantly changed proteins (NC proteins) and are represented as gray dots.

For bioinformatics analysis, the LC-MS/MS data were analyzed using the Thermo Fisher Scientific Proteome Discover software suite 2.2 with the SEQUEST search engine. Data were compared with those obtained from the rhesus macaque unreviewed FASTA database downloaded from UniProt (released on July 21, 2017). Significantly changed proteins were identified using the following thresholds: downregulation, 0.67 (2^−0.58^) and upregulation, 1.50 (2^0.58^). The scatter graph was drawn using the ggplot2 package from the Bioconductor R toolset. The heatmap was generated using HemI software. Gene ontology (GO) analysis was performed using PANTHER^1^. Pathway analysis was performed using the Wiki pathway database of the WEB-based Gene SeT AnaLysis Toolkit^2^ (see workflow in Figure 1A). The STRING (Search Tool for the Retrieval of Interacting Genes/Proteins) database^3^ was used for predicting protein networks. All STRING network analyses were performed using protein accessions as input and with the confidence level threshold set at high (0.7).

The mass spectrometry proteomics data have been deposited with the ProteomeXchange Consortium *via* the PRIDE (Vizcaíno et al., 2016) partner repository under the dataset identifier PXD012306.

### Western Blot

Protein concentrations were determined using a NanoDrop 2000 (Thermo Fisher Scientific, NJ, USA). Ten microgram of tissue lysates in each sample were loaded into 10% polyacrylamide gels for SDS-PAGE, and transferred onto nitrocellulose membranes. The membranes were then blocked with 5% non-fat dry milk in TBS-T (Tris Buffered Saline plus 0.5% Tween) for 40 min and incubated with primary antibodies (1:10,000 for ACTB, GTX124213, GeneTex; 1:1,000 for FN1, AB32419, Abcam; 1:1,000 for NOS3, SC-376751, Santa Cruz, CA, USA) overnight at 4°C. Horseradish peroxidase-conjugated secondary antibodies were used to visualize protein bands. Band intensity was analyzed after incubation with ECL reagents, and imaged.

### Immunohistochemistry (IHC)

The 10-μm frontal cortex slices were obtained using a freezing microtome (Leica, Nussloch, Baden-Württemberg, Germany). Brain slices were pretreated for peroxidase activity using 3% hydrogen peroxide (H_2_O_2_) in 0.1 M PBS containing 5% Triton X-100 for 20 min, and blocked with 5% normal horse serum (NHS; Vector Laboratories, Burlingame, CA, USA) for 1 h. Thereafter, selected tissue slices were incubated with primary antibody of beta-amyloid Clone 6F/3D (1:800, Code-Nr. M 0872, Dako) at 4°C overnight. Slices were again washed three times with 0.1 M PBS, incubated with biotinylated horse anti-rabbit IgG (Vector Laboratories, Burlingame, CA, USA) secondary antibody for 1 h, and then treated with biotinylated protein A and avidinbiotinylated horseradish peroxidase complexes (ABC kit, Vector Laboratories, Burlingame, CA, USA) for another 1 h at room temperature. Immunoreactive cells were visualized using 3,3′-diaminobenzidine (DAB) and urea H_2_O_2_ tablets (Sigma-Aldrich, St. Louis, MO, USA) dissolved in double distilled water. Finally, sections were mounted onto glass slides, dehydrated with a graded series of ethanol, cleared in xylene, and cover slipped.

### Statistical Analysis

The difference of Western blot data between two groups was analyzed by two-tailed Student’s *t*-test. A *p*-value of less than 0.05 was defined as the threshold for statistical significance.

## Results

### Sample Information

In this study, cerebral vessels were resected from rhesus macaques after deep euthanasia. Vessel specimens were divided into three groups: Group A (age = 3 years, two males and one female), Group B (age = 6 years, one male and two females), Group C (age = 20 years, two males and one female). No obvious senile plaques were detected (Supplementary Figure S1). No neurological diseases or other diseases were found in these rhesus macaques.

### Quantitative MS Analysis of Protein Profiles

To analyze the proteomic changes associated with cerebrovascular aging, the soluble tissue extracts were TMT-labeled for LC-MS/MS analysis. A total of 2,934 proteins were identified (unique peptides ≥2, false discovery rate (FDR) ≤0.01, Supplementary Table S1). Comparison of Group B with Group A revealed 57 (1.9%) upregulated proteins and 272 (9.3%) downregulated proteins (129/127 ratio ≥1.50, or ≤0.67), while comparison of Group C with Group B showed 96 (3.3%) upregulated proteins and 321 (10.9%) downregulated proteins (130/129 ratio ≥1.50, or ≤0.67, Figure 1B). All the significantly changed proteins detected are displayed in Supplementary Table S1.

**Figure 2:**
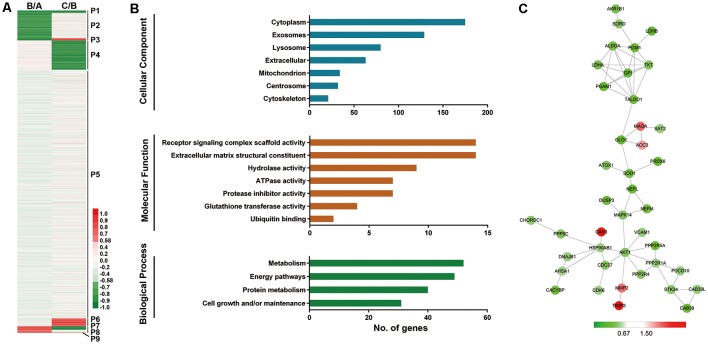
Profiling of proteins significantly related to aging (SA proteins). ** (A)** Heatmap for identification of SA proteins. Twenty-two proteins were continuously downregulated during aging (P1); 270 proteins were downregulated after 6-year old (P4); 73 proteins were upregulated after 6-year old (P6); three proteins were continuously upregulated with age (P9). These proteins (P1+P4+P6+P9, 368 proteins) are thought to be significantly related to aging (SA proteins). **(B)** Gene ontology (GO) analysis revealed protein classification of SA proteins using PANTHER (http://www.pantherdb.org/). **(C)** Protein-protein interactions with STRING analysis. For each identified protein, the 130/129 ratio (Group C/Group B) is shown. Proteins related to energy pathway (such as GPI and ALDOA), superoxide metabolism (such as SOD1 and PRDX6), and serine/threonine kinase (such as AKT1 and STK24) served as the hubs in the networks.

In comparisons of Group B vs. Group A and Group C vs. Group B, 22 proteins were downregulated; thus, these proteins were continuously downregulated during aging (P1, Figure 2A). There were 270 proteins that were not changed when comparing Group B vs. Group A, but were found to be downregulated when comparing Group C vs. Group B. These proteins were downregulated after 6-year old (P4, Figure 2A). In addition, 73 proteins were upregulated after 6-year old (P6, Figure 2A) and three proteins were upregulated continuously with age (P9, Figure 2A).

We next performed GO analysis of proteins P1, P4, P6, and P9, which were thought to be significantly related to aging (SA proteins, 368 proteins, shown in Supplementary Table S2). UniProt accession profiles of these SA proteins were classified using PANTHER^4^ (Figure 2B). Most of the SA proteins studied were from cytoplasm (64.8%), exosome (47.8%) and lysosome (29.6%). Receptor signaling complex scaffold activity (5.2%) and ECM structural constituent (5.2%) were the most prominent molecular functions of the proteins. In terms of biological process, the altered proteins were involved mainly in metabolism (19.3%), energy pathways (18.1%), and protein metabolism (14.8%).

To further understand the protein-protein interactions among the identified SA proteins P1, P4, P6, and P9 during normal vascular aging, STRING tools were used to create comprehensive networks with the confidence level threshold set at high (0.7; Figure 2C). The most connected proteins were related mainly to energy pathways (such as GPI and ALDOA), superoxide metabolism (such as SOD1 and PRDX6), and serine/threonine kinases (such as AKT1 and STK24). Interestingly, most proteins in the network were downregulated when comparing Group C vs. Group B.

### Pathway Analysis

**Figure 3:**
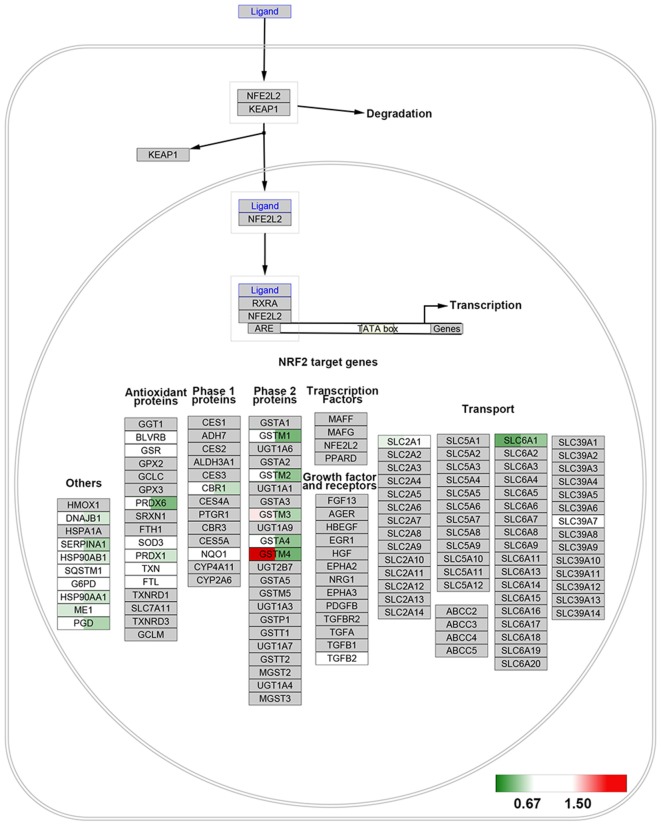
NRF2 pathway. All identified proteins were mapped to the NRF2 pathway based on the published WikiPathway database. For each identified protein, the 129/127 ratio (Group B/Group A) is displayed on the left, and the 130/129 ratio (Group C/Group B) is shown on the right. The overexpressed proteins are shown with a red background and the downregulated proteins are shown with a green background.

**Figure 4:**
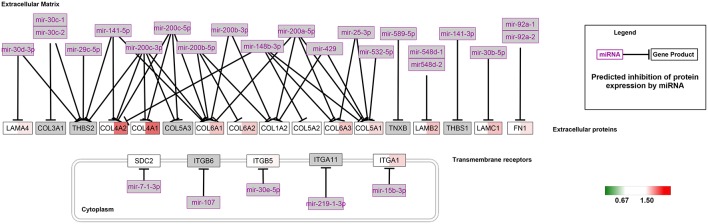
MiRNA targets in the extracellular matrix (ECM) and membrane receptors pathway. For each identified protein, the 129/127 ratio (Group B/Group A) is displayed on the left, and the 130/129 ratio (Group C/Group B) is shown on the right. The overexpressed proteins are shown with a red background and the downregulated proteins are shown with a green background.

The Wiki pathway database of the WEB-based Gene SeT AnaLysis Toolkit was employed to clarify the biological pathways involved in aging. Of the 368 SA proteins, 272 were successfully matched to the homologous human proteins using bioDBnet^5^ (Supplementary Table S2) and included in the pathway analysis. The top 10 enriched pathways listed in Supplementary Table S3 included the NRF2 pathway and miRNA targets in the ECM and membrane receptors. Twenty-six proteins were identified in the NRF2 pathway (Figure 3) and 15 proteins in miRNA targets in the ECM and membrane receptors (Figure 4). Interestingly, almost all SA proteins in the NRF2 pathway were downregulated when comparing Group C vs. Group B, including DNAJB1, SERPINA1, HSP90AB1, HSP90AA1, PGD, PRDX6, PRDX1, CBR1, GSTM1, GSTM2, GSTM3, and GSTA4. In addition, SLC6A1 was continuously downregulated. In contrast, in the miRNA targets in the ECM and membrane receptors pathway, all SA proteins were upregulated when comparing Group C vs. Group B, including LAMA4, LAMB2, LAMC1, COL4A1, COL4A2, COL6A1, COL6A2, COL6A3, COL5A1, FN1, and ITGA1.

### Verification of Protein Expression Levels

**Figure 5:**
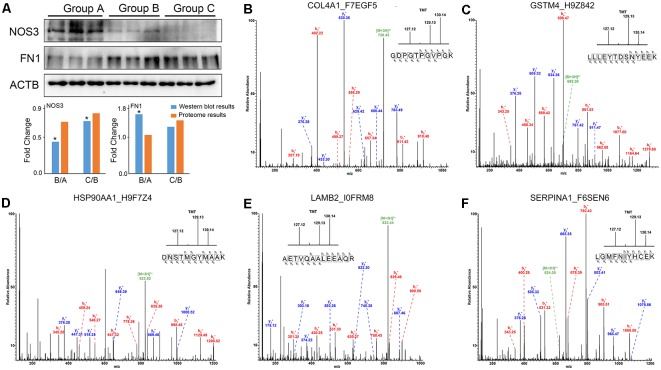
The changes in protein expression were confirmed by Western blot and MS/MS analyses. ** (A)** Western blot analysis of NOS3, FN1 and ACTB proteins from Group A, B, and C. The results were consistent with the proteomics data. *p < 0.05 (two-tailed Student’s *t*-test). **(B–F)** The representative MS/MS spectrum data of COL4A1, GSTM4, HSP90AA1, LAMB2, SERPINA1. The column height of the TMT diagram indicates the relative quantification of the peptide segment in Group A, B, C. 127.12 (TMT-127) for Group A, 129.13 (TMT-129) for Group B, and 130.14 (TMT-130) for Group C.

To validate the reliability of the quantitative proteomic analysis, the individual protein sample from Group A, B and C was subjected to Western blot analysis. Two proteins (NOS3, FN1) were identified in this study. There was a high level of similarity in the binding sequences of the anti-NOS3, anti-FN1 and anti-ACTB antibodies for human and rhesus macaque NOS3, FN1 and ACTB (Supplementary Figure S2). Significant downregulation of NOS3 (Group B/Group A and Group C/Group B) and upregulation of FN1 (Group B/Group A) were found with increasing age (*p* < 0.05). The fluctuation in the levels of these proteins was consistent with those observed in the proteomics data (Figure 5A). The representative MS/MS spectrum data for COL4A1, GSTM4, HSP90AA1, LAMB2, and SERPINA1 are displayed for validation of the proteomics data in Figures 5B–F.

## Discussion

In the present study, we used proteomics analysis to identify 2,934 proteins expressed in cerebral veins of rhesus macaques. A total of 329 proteins were differentially expressed from 3-year old to 6-year old, while 417 were differentially expressed after 6-year old in rhesus macaques. Most of these changed proteins were downregulated during aging. Moreover, our results revealed that protein expression in a variety of molecular pathways, such as the NRF2 pathway (Figure 3) and miRNA targets in the ECM and membrane receptors (Figure 4), was significantly changed during aging.

Structural and functional changes in the vascular endothelium and the tunica media were the main causes of cerebrovascular aging (Izzo et al., 2018). According to recent studies, dysfunction of the vascular endothelium is related to disorders of energy metabolism and oxidative stress (Donato et al., 2007; Valko et al., 2007; Ungvari et al., 2010a; Izzo et al., 2018). Moreover, physical and pathological changes in the tunica media, such as increased calcium deposits, accumulating ECM, and hypertrophy, increase vascular stiffness in the elderly (Zieman et al., 2005; Izzo et al., 2018).

### Age-Related Endothelial Dysfunction

An imbalance between the production and elimination of ROS may result in oxidative stress. ROS accumulation causes inflammation and the accumulation of misfolded proteins which indirectly damage the vessel wall. ROS also directly oxidizes the cytoplasmic membrane, leading to endothelial dysfunction (Förstermann et al., 2017).

Proteins related to energy metabolism, such as GPI, ALDOA, and TKT, were highly connected in the protein-protein interaction networks, and were mostly downregulated when comparing Group C vs. Group B, which was consistent with the results of GO biological process analyses. ROS are produced mainly in the mitochondria *via* mitochondrial respiration (Ungvari et al., 2010b; Izzo et al., 2018). Disorders in energy pathways can contribute to the over-production of ROS in the mitochondria, causing damage to mitochondrial constituents and leading to further ROS production (Beckman and Ames, 1998).

Moreover, our results indicated that a variety of the changed proteins were related to the NRF2 pathway, and most were downregulated after 6-year old (Figure 2). The NRF2 pathway plays a major role in cellular antioxidative activity (Nguyen et al., 2009). It has been reported that ROS-induced endothelial damage is mediated by suppression of the NRF2 pathway, leading to a reduction in antioxidant defense against ROS (Dai et al., 2012; Meng et al., 2017). The protein-protein interaction results also indicated that some proteins, such as SOD1 and PRDX6, were related to superoxide metabolism and served as hubs in the networks (Figure 4); all of these proteins were downregulated when comparing Group C vs. Group B.

Glutathione S-transferase Mu (GSTM) is one of the key proteins in the NRF2 pathway. In this study, we identified four (GSTM1, GSTM2, GSTM3, and GSTM4) out of five GSTM subunit types in the brain vessels and all four were significantly downregulated when comparing Group C vs. Group B. GSTMs catalyze nucleophilic addition and substitution reactions utilizing GSH to detoxify a wide variety of electrophilic substrates, such as ROS (Patskovsky et al., 2006), and thus, are critical for the antioxidant bioactivity of the NRF2 pathway. Previous studies demonstrated that the ability of ROS to promote NRF2 translocation and induce GSTM expression reduces with age in *Caenorhabditis elegans*, human fibroblasts (Meng et al., 2017), and Asian clam (Vrankovic, 2016). Furthermore, GSTM3 has been reported to protect the lens from oxidative stress to prevent age-related cataract formation in humans (Li et al., 2016). Our results are the first to demonstrate the relationship between downregulation of GSTM expression and cerebrovascular aging.

Thus, increased ROS production in aging vessels inhibits activation of the NRF2 pathway, especially *via* GSTM, leading to increased sensitivity to oxidative damage (Ungvari et al., 2008, 2011a,b). This phenomenon could be closely associated with the age-related endothelial dysfunction.

### Age-Related Tunica Media Changes

GO analysis showed that most of the altered proteins were structural constituents of the ECM. The main components of the ECM of the vessel wall are collagen, elastin, glycoproteins and proteoglycans. The collagen and elastin of the tunica media provide the structural integrity and elasticity of the majority of arteries. Many previous studies demonstrated that altered expression of collagen and elastin in the ECM of vascular smooth muscle cells in the tunica media leads to increased vascular stiffness and atherosclerosis (Lacolley et al., 2017). Our results indicate that some ECM proteins, such as type IV collagen alpha-1 chain (COL4A1) and type IV collagen alpha-2 chain (COL4A2), were significantly upregulated when comparing Group C vs. Group B. In addition, AKT1, the serine/threonine protein kinase, which has a pivotal role in preventing programmed cell death (Dudek et al., 1997), was found to be most connected in the protein-protein interaction networks, and was significantly downregulated. Thus, collagen overexpression and increased programmed cell death might be major contributors to age-related vascular stiffness.

In our study, we found that the expression of seven proteins was significantly altered in miRNA targets in the ECM and membrane receptors, all of which were upregulated (Group C/Group B; Figure 3), including COL4A1 and COL4A2. Since COL4A1 and COL4A2 are suppressed by miRNAs, the alterations in miRNAs might be closely related to age-related cerebrovascular stiffness. Recently, an increasing number of studies have shown that alterations in miRNA are associated with age-related physiological and functional disorders of vessels (Lin et al., 2016; Lacolley et al., 2017). For example, miRNA-mediated regulation of vascular smooth muscle cell contractile proteins was the basic mechanism leading to arterial stiffening and decreased compliance in mice (Cui et al., 2012; Lacolley et al., 2017). Furthermore, Paladino et al. (2018) reported that miRNAs directly regulate the NRF2 pathway, leading to the alterations in antioxidant bioactivity in vessels. In the present study, our results support the critical role of miRNAs in cerebrovascular aging.

Compared to traditional rodent animals, non-human primates show more similarities with humans, especially in brains. Thus, studies based on rhesus tissues are highly important, especially those focusing on central nervous systems. Specimens from rhesus macaques were used in this study instead of humans due to the difficulties in obtaining brain vessel biopsies from a healthy human using non-invasive methods. However, only limited rhesus macaques in each group were available in our study, and thus the results of this study should be interpreted with caution. Studies using a greater number of specimens from rhesus macaque or human brain vessels are needed in the future.

## Conclusion

Our study demonstrates that cerebrovascular aging involves many pathways, including the NRF2 pathway and miRNA targets in the ECM and membrane receptors. Cerebrovascular aging is exacerbated by the over-production of ROS caused by disorders of energy metabolism and dysfunction of antioxidant activity caused by suppression of the NRF2 pathway and downregulation of GSTM protein expression, especially after 6-year old. Furthermore, miRNA-regulated accumulation of ECM proteins might be closely associated with age-related arterial stiffening and decreased compliance.

## Data Availability Statement

The datasets generated for this study can be found in the proteomics data available *via* ProteomeXchange with the identifier PXD012306.

## Ethics Statement

The animal study was reviewed and approved by the Ethical Review Committee of the Chinese Academy of Medical Sciences.

## Author Contributions

XW, WG and ZH designed the research. XW, YJ and HL performed the research. XW, YL and XB analyzed the data. The manuscript was written through the contributions of all authors. All authors have given approval to the final version of the manuscript.

## Conflict of Interest

The authors declare that the research was conducted in the absence of any commercial or financial relationships that could be construed as a potential conflict of interest.

## Footnotes

^1^http://www.pantherdb.org/

^2^http://www.webgestalt.org

^3^http://string-db.org

^4^http://www.pantherdb.org/

^5^https://biodbnet-abcc.ncifcrf.gov

## Supplementary Material

FIGURE S1 Immunostaining of beta-amyloid in the frontal cortex of three individual rhesus macaques. No obvious senile plaques were detected in three groups of monkeys.

FIGURE S2 The binding sequences of anti-NOS3, anti-FN1 and anti-ACTB antibodies showed a high level of similarity between human and rhesus macaque NOS3, FN1 and ACTB. **(A)** Protein sequence alignment of ACTB (Human and Rhesus macaque), and anti-ACTB information. **(B)** Protein sequence alignment of NOS3 (Human and Rhesus macaque), and anti-NOS3 information. **(C)** Protein sequence alignment of FN1 (Human and Rhesus macaque), and anti-FN1 information.

TABLE S1 All identified proteins and significantly changed proteins when comparing Group B to Group A and Group C to Group B.

TABLE S2 A total of 368 proteins significantly related to aging (SA proteins).

TABLE S3 Pathway list generated by the analysis of SA proteins.
