# Supplementary material for: Proteome Profiling of Cerebral Vessels in Rhesus Macaques: Dysregulation of Antioxidant Activity and Extracellular Matrix Proteins Contributes to Cerebrovascular Aging in Rhesus Macaques
Source: Front Aging Neurosci. 2019 Oct 23;11:293. doi: 10.3389/fnagi.2019.00293 (PMC6819311; doi:10.3389/fnagi.2019.00293)
Supplement: TABLE S1 — All identified proteins and significantly changed proteins when comparing Group B to Group A and Group C to Group B. [file Table_3.DOCX]

**Supplementary Table 3. Pathway list generated by the analysis of SA proteins.**

| Pathway Name | #Gene | EntrezGene | Statistics |
| --- | --- | --- | --- |
| miRNA targets in ECM and membrane receptors | 7 | 1282 1284 1291 1292 1293 3913 3915 | C=22; O=7; E=0.65; R=10.82; PValue=1.94e-06; FDR=3.91e-04 |
| Composition of Lipid Particles | 5 | 335 336 338 345 348 | C=9; O=5; E=0.26; R=18.9; PValue=2.35e-06; FDR=3.91e-04 |
| Statin Pathway | 6 | 335 336 338 345 348 5360 | C=29; O=6; E=0.85; R=7.04; PValue=1.58e-04; FDR=1.75e-02 |
| Alpha 6 Beta 4 signaling pathway | 6 | 1432 207 2549 3913 3915 5781 | C=33; O=6; E=0.97; R=6.18; PValue=3.35e-04; FDR=2.78e-02 |
| PPAR Alpha Pathway | 5 | 1622 335 336 345 5360 | C=26; O=5; E=0.76; R=6.54; PValue=8.2e-04; FDR=5.44e-02 |
| NRF2 pathway | 12 | 2941 2944 2946 2947 2948 3320 3326 5052 5265 6529 873 9588 | C=145; O=12; E=4.26; R=2.81; PValue=1.04e-03; FDR=5.76e-02 |
| Vitamin B12 Metabolism | 6 | 3043 335 338 3383 348 6647 | C=50; O=6; E=1.47; R=4.08; PValue=3.19e-03; FDR=1.32e-01 |
| Hepatitis C and Hepatocellular Carcinoma | 6 | 1284 1432 207 5420 5781 7345 | C=50; O=6; E=1.47; R=4.08; PValue=3.19e-03; FDR=1.32e-01 |
| Glutathione metabolism | 4 | 2687 290 2944 2946 | C=23; O=4; E=0.68; R=5.92; PValue=4.11e-03; FDR=1.42e-01 |
| Interferon type I signaling pathways | 6 | 10399 1398 1432 1975 5781 6772 | C=54; O=6; E=1.59; R=3.78; PValue=4.71e-03; FDR=1.42e-01 |

•C: the number of reference genes in the category

•O: the number of genes in the gene set and also in the category

•E: the expected number in the category

•R: ratio of enrichment
