# Supplementary figures and images for: Proteome Profiling of Cerebral Vessels in Rhesus Macaques: Dysregulation of Antioxidant Activity and Extracellular Matrix Proteins Contributes to Cerebrovascular Aging in Rhesus Macaques
Source: Front Aging Neurosci. 2019 Oct 23;11:293. doi: 10.3389/fnagi.2019.00293 (PMC6819311; doi:10.3389/fnagi.2019.00293)

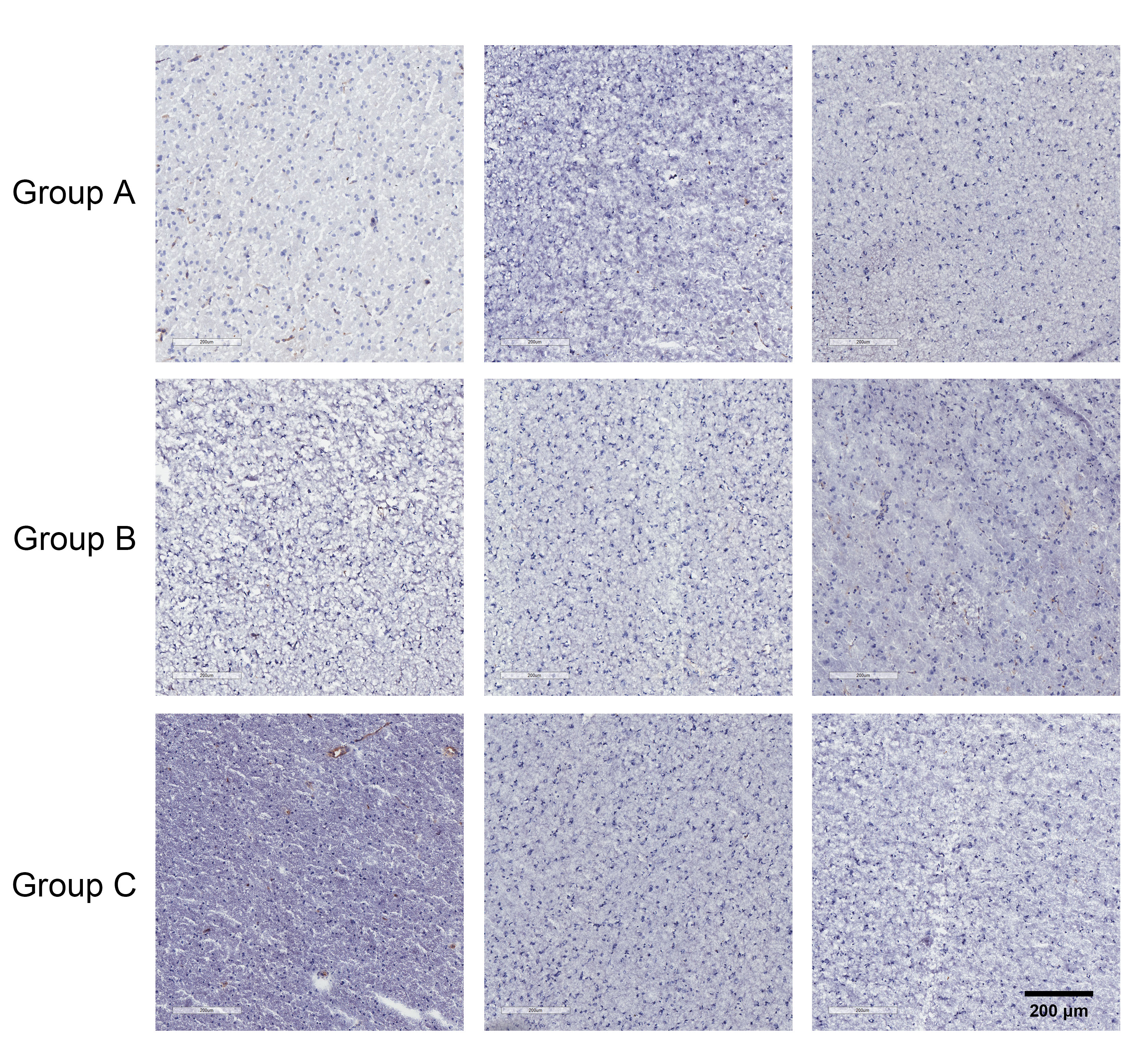

Supplement: TABLE S2 — A total of 368 proteins significantly related to aging (SA proteins). [file Image_1.JPEG]

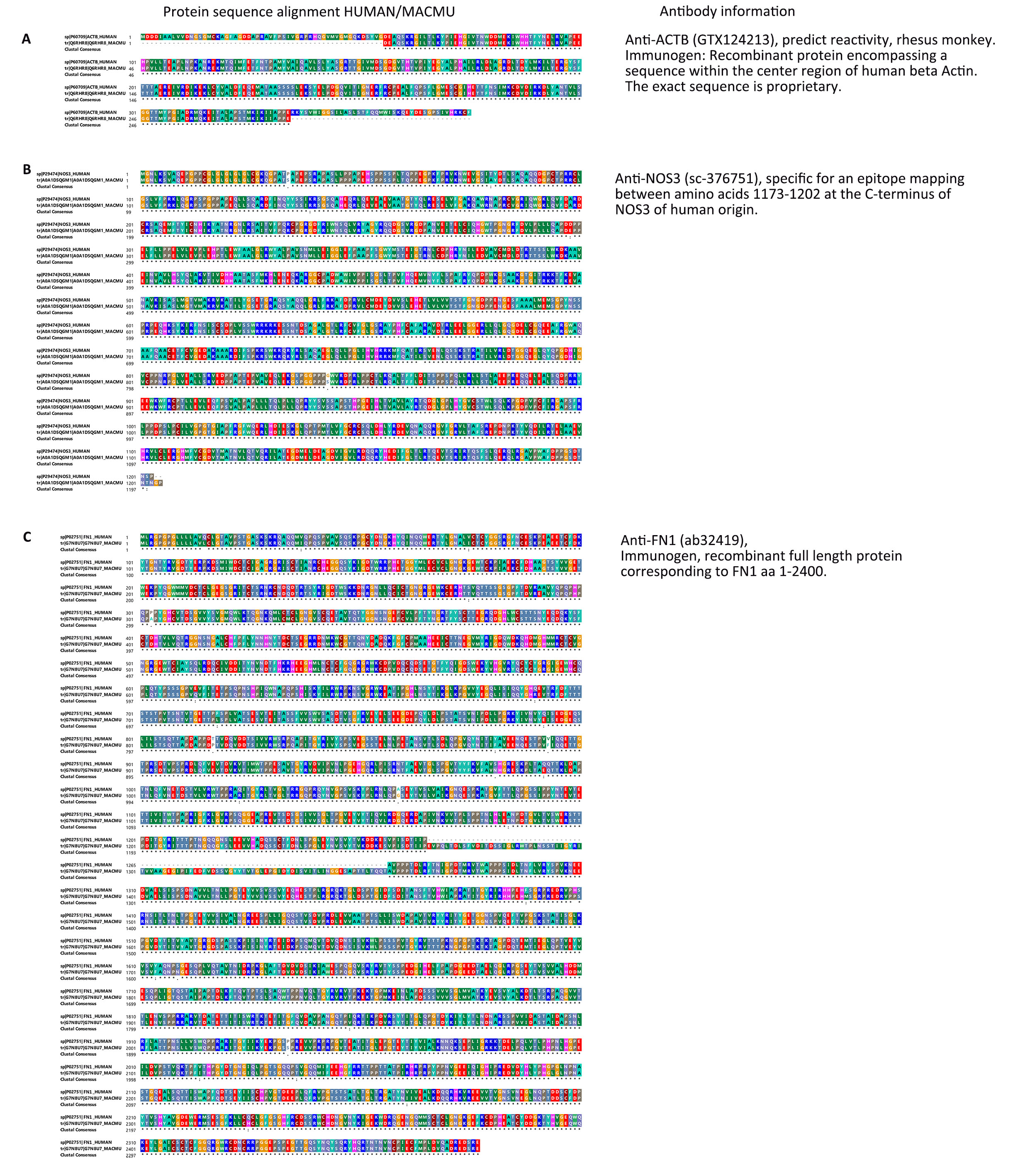

Supplement: TABLE S3 — Pathway list generated by the analysis of SA proteins. [file Image_2.JPEG]
